# Antioxidant, Anti-Inflammatory, and Multidrug Resistance Modulation Activity of Silychristin Derivatives

**DOI:** 10.3390/antiox8080303

**Published:** 2019-08-14

**Authors:** Jitka Viktorová, Simona Dobiasová, Kateřina Řehořová, David Biedermann, Kristýna Káňová, Karolína Šeborová, Radka Václavíková, Kateřina Valentová, Tomáš Ruml, Vladimír Křen, Tomáš Macek

**Affiliations:** 1 Department of Biochemistry and Microbiology, University of Chemistry and Technology Prague, Technická 5, CZ 166 28 Prague, Czech Republic; 2 Laboratory of Biotransformation, Institute of Microbiology, Czech Academy of Sciences, Vídeňská 1083, CZ 142 20 Prague, Czech Republic; 3 Toxicogenomics Unit, National Institute of Public Health, Šrobárova 49, CZ 100 00 Prague, Czech Republic; 4 Laboratory of Pharmacogenomics, Biomedical Center, Faculty of Medicine in Pilsen, Charles University, alej Svobody 1655, CZ 323 00 Pilsen, Czech Republic

**Keywords:** Adriamycin, P-glycoprotein, silymarin, silychristin, immunomodulation, ABC superfamily, BCRP, expression profile

## Abstract

Silychristin A is the second most abundant compound of silymarin. Silymarin complex was previously described as an antioxidant with multidrug resistance modulation activity. Here, the results of a classical biochemical antioxidant assay (ORAC) were compared with a cellular assay evaluating the antioxidant capacity of pure silychristin A and its derivatives (anhydrosilychristin, isosilychristin and 2,3-dehydrosilychristin A). All the tested compounds acted as antioxidants within the cells, but 2,3-dehydro- and anhydro derivatives were almost twice as potent as the other tested compounds. Similar results were obtained in LPS-stimulated macrophages, where 2,3-dehydro- and anhydrosilychristin inhibited NO production nearly twice as efficiently as silychristin A. The inhibition of P-glycoprotein (P-gp) was determined in vitro, and the respective sensitization of doxorubicin-resistant ovarian carcinoma overproducing P-gp was detected. Despite the fact that the inhibition of P-gp was demonstrated in a concentration-dependent manner for each tested compound, the sensitization of the resistant cell line was observed predominantly for silychristin A and 2,3-dehydrosilychristin A. However, anhydrosilychristin and isosilychristin affected the expression of both the P-gp (*ABCB1*) and *ABCG2* genes. This is the first report showing that silychristin A and its 2,3-dehydro-derivative modulate multidrug resistance by the direct inhibition of P-gp, in contrast to anhydrosilychristin and isosilychristin modulating multidrug resistance by downregulating the expression of the dominant transmembrane efflux pumps.

## 1. Introduction

**Figure 1:**
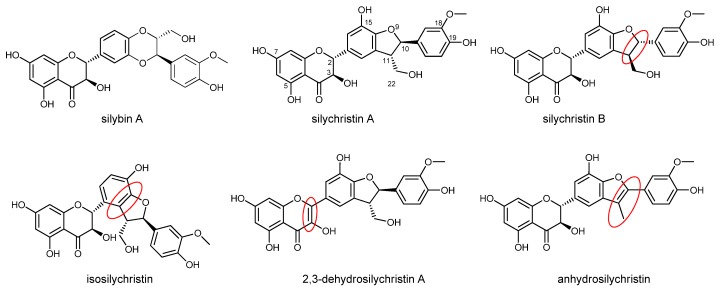
Structure of silychristin diastereomers and derivatives. Major structural differences from silychristin A are highlighted with red ovals. The structure of silybin A is shown for comparison.

Silychristin is the second most abundant flavonolignan in the silymarin complex, which is usually produced by the acetone extraction of *Silybum marianum* (L.) Gaertn. (milk thistle) fruits [1]. Despite its high content in the silymarin complex and its biological potential, little attention has been paid to this compound, mainly due to its rather difficult separation leads to its co-elution with one of its isomeric flavonolignans, silydianin [1], a problem that has been resolved recently [2] by careful chromatography on LH-20 gel. Natural silychristin is a mixture of two diastereomers (silychristin A and B, 95:5). Isosilychristin is a silychristin isomer that mainly occurs in wild milk thistle, while 2,3-dehydrosilychristin is a product of its aerial oxidation [3,4] detectable in silymarin preparations. In contrast, anhydrosilychristin is a dehydrated product obtained by the treatment of silychristin with hydrochloric acid in hot ethanol [1] (Figure 1). The biological potential of these derivatives has been reviewed recently [1].

As other components of the silymarin complex, silychristin has antioxidant properties. Its potential to scavenge the 2,2-diphenyl-1-picrylhydrazyl (DPPH) radical is nearly 14× higher than that of silybin (considered “the active component of silymarin”) and approximately 1.5× lower than that of its oxidized derivative 2,3-dehydrosilybin [5,6]. Moreover, silychristin exhibited a higher antioxidant capacity than “traditional” antioxidants such as synthetic phenolic antioxidants: butylated hydroxyanisole (BHA), butylhydroxytoluene (BHT), α-tocopherol and trolox [7]. As the first-pass metabolism of polyphenols such as silychristin is usually connected to conjugation [8], its sulfated derivatives were also tested for their antioxidant properties. Although the sulfated metabolite of silychristin was a less potent antioxidant, sulfated 2,3-dehydrosilychristin was more active in FCR (Folin–Ciocalteu reagent reduction) and FRAP (ferric reducing antioxidant power) assays than the parent compound [9].

Besides antioxidant activity, several other biological activities have been reported for silychristin. Silychristin was able to inhibit α-glucosidase [10], exhibiting a potential in the treatment of diabetes mellitus type II. Furthermore, silychristin increased insulin secretion and decreased glucose content in induced type I diabetic rats [10] and *Mesocestoides vogae* larvae [11]. Silychristin also displayed concentration-dependent anti-inflammatory activity [12] and inhibited collagenase much more efficiently than its standard inhibitor 1,10-phenanthroline [5], thus showing a potential application in cosmeceuticals. In a transdermal study, silychristin penetrated into the human skin but did not reach the basolateral side [13]. Both acute cytotoxic and genotoxic doses were higher than 100 µM for blood platelets, peripheral blood mononuclear cells, and alveolar basal epithelial cells. Moreover, at these concentrations, silychristin protected mitochondria against spontaneous DNA damage [14]. Moreover, copper and iron chelation was affected by silychristin as well, with possible implications for the absorption of these ions in the gastrointestinal tract [15].

The antithrombotic activity was described in detail by the group of Bijak et al.; silychristin inhibited ADP-induced blood platelet activation via the G protein-coupled receptor P2Y12. This blockade could potentially reduce the peripheral artery disease, myocardial infarction, ischemic stroke, and vascular death [16]. Similarly, silychristin inhibited collagen-induced blood platelet activation, the usual response to tissue injury, which can lead to thrombotic events in cases of overactivation [17].

In addition to α-glucosidase, silychristin has also been described as an inhibitor of human carbonic anhydrase. This enzyme is responsible for maintaining the acid‒base balance and for the transport of carbon dioxide. Inhibitors of this enzyme have several clinical applications, as a diuretic and antiepileptic, and in the treatment of gastric and duodenal ulcers, but their primary use is in the treatment of glaucoma [18].

In this study, the antioxidant, anti-inflammatory, and MDR modulation activities of silychristin are compared to its natural and synthetic derivatives. We demonstrate that 2,3-dehydrosilychristin is the most promising antioxidant and anti-inflammatory compound. Moreover, we describe the ability of silychristin and its 2,3-dehydro- derivative to inhibit P-glycoprotein (P-gp) and thus reverse the doxorubicin-resistance phenotype in resistant human ovarian carcinoma. In contrast to this direct inhibition of P-gp, we suggest that anhydrosilychristin and isosilychristin modulates the multidrug resistance by downregulating the expression of the dominant transmembrane efflux pumps.

## 2. Materials and Methods

### 2.1. Analytical Standards and Chemicals

We used 2,2′-azo-*bis-*(2-methylpropionamidine) dihydrochloride (AAPH, Sigma-Aldrich, St. Louis, MO, USA); 2′,7′-dichlorofluorescin diacetate (DCFH-DA, Sigma-Aldrich); 100× antibiotic antimycotic solution (Sigma-Aldrich); fetal bovine serum (FBS, Sigma-Aldrich); Eagle’s minimum essential medium (EMEM, Sigma-Aldrich); lipopolysaccharides from *Escherichia coli* O111:B4 (LPS, Sigma-Aldrich); fluorescein (Sigma-Aldrich); Griess reagent modified (Sigma-Aldrich); resazurin sodium salt (Sigma-Aldrich); Pgp-Glo Assay System (Promega, Madison, WI, USA); doxorubicin hydrochloride (sold under the trade name Adriamycin, Sigma-Aldrich); Trizol Reagent (Thermo Fisher Scientific, Walham, MA, USA); l-glutamine solution (Sigma-Aldrich); trypsin‒EDTA solution (Sigma-Aldrich); Essential Medium Eagle no phenol red (MEM, Sigma-Aldrich); and Dulbecco’s Modified Eagle’s medium—high glucose (DMEM, Sigma-Aldrich). Silybin was isolated from silymarin by its quick suspension in methanol and filtration, yielding solid silybin A+B. Silybin diastereomers were isolated as described previously [19] to obtain silybin A (99%) and silybin B. Briefly, silybin diastereomers are chemoenzymatically resolved by immobilized lipase B from *Candida antarctica* (Novozyme 435, Novo-Nordisk, Copenhagen, Denmark). A series of consecutive acetylations and solvolyses was used. Silychristin A (96.4%, containing 3.6% of silychristin B) was isolated from silymarin by Sephadex LH-20 chromatography as described in [2]. 2,3-Dehydrosilychristin A (91.2%, containing 8.8% of silychristin B), isosilychristin (95.8%) and anhydrosilychristin (92%, containing ca. 4% of silychristin A) were prepared as described by Biedermann et al. [1]. Briefly, 2,3-dehydrosilychristin A was synthesized by oxidation of silychristin with gaseous oxygen in DMSO in the presence of triethylamine; anhydrosilychristin was prepared from silychristin by reflux in HCl/EtOH and isosilychristin was isolated from silymarin by chromatography on an ASAHIPAK GS-310 20f column (Showa Denko K. K., Tokyo, JP). The NMR and MS spectra of compounds used were identical to the authentic standards available in the Laboratory of Biotransformation, Institute of Microbiology, CAS, Prague [1].

### 2.2. Antioxidant Capacity

An oxygen radical absorption capacity (ORAC) assay was performed according to [20]. Briefly, a stock solution of fluorescein (0.44 mg/mL) was prepared in phosphate-buffered saline (PBS, pH 7.4) and stored in a freezer (−20 °C) wrapped in aluminum foil. Prior to use, 167 µL of the stock solution was diluted with 25 mL of PBS. Using a dispenser (MultiFlo Microplate Dispenser, BioTek Instruments, Winooski, VT, USA), 50 µL of this solution was split into the wells of a 96-well plate. The concentration range (1.25–20 µM) of the samples was prepared by the binary dilution of the samples. Two microliters of the samples and 23 µL of PBS were added to the wells. After 15 min of incubation (37 °C), 25 µL of freshly prepared AAPH (60 mg/mL) was added to each well except for the negative control, where AAPH was replaced with PBS. Immediately after the addition, the fluorescence (ex./em., 485/535 nm) was recorded for 2 h with a measurement step of 5 min using a microplate reader (SpectraMax i3 Multi-Mode Detection Platform, Molecular Devices, San Jose, CA, USA).

For the cellular antioxidant activity (CAA) assay, the HepG2 cell line (ATCC, CCL-23TM, Manassas, VA, USA) was cultivated in EMEM supplemented with 10% of FBS, 2 mM l-glutamine and 1× Antibiotic Antimycotic Solution. The cells were cultivated in a CO_2_ incubator (5% CO_2_, 37 °C, Thermo Fisher Scientific) and passaged twice per week according to a standardized protocol using a trypsin‒EDTA solution. For the experiments, 100 µL of the cells with density corresponding to 1 × 10^6^ cells/mL (Cellometer Auto T4 Bright Field Cell Counter, Nexcelom Bioscience, Lawrence, MA, USA) were split into 96-well plates. After 24 h, the cells were washed 3× with PBS (MultiFlo Multi-Mode Dispenser, BioTek) and DMEM supplemented with the DCFH-DA (0.0125 mg/mL) was added to each well together with the tested samples in the concentration range 0.5–2.25 µM. After 1 h incubation in a CO_2_ incubator, the medium was manually replaced with AAPH solution (0.16 mg/mL in PBS) and the fluorescence was immediately recorded (ex./em. 485/540 nm) for 2 h in 5-min steps. This protocol was slightly modified version of a published procedure [21].

### 2.3. Anti-Inflammatory Properties

Macrophages (RAW 264.7, Sigma-Aldrich) were cultivated in DMEM supplemented with 2 mM l-glutamine and 1× antibiotic antimycotic solution, similarly to the HepG2 cells. However, this cell line was not detached by the trypsin‒EDTA solution, but mechanically with a scraper. For the experiment, the cells were seeded at 1 × 10^6^ cells/mL into the 96-well plates. After 48 h, the cells were washed 3× with PBS, LPS (1 µg/mL in MEM) and the samples in the concentration range of 6.25–100 µM were added to a final volume of 100 µL. After 24 h, the medium was mixed with Griess reagent (0.04 g/mL, prepared freshly in deionized water, Sigma-Aldrich) in a 1:1 ratio in a new 96-well plate. The absorbance was measured at 540 nm after 15 min. The cells were incubated with resazurin (0.03 mg/mL in PBS) for 2 h, after which the fluorescence was recorded (ex./em. 560/590 nm).

### 2.4. Inhibition of P-Glycoprotein

The *in vitro* inhibition of P-gp was tested using the Pgp-Glo Assay System according to the manufacturer’s instructions. Briefly, the reaction mixture contained Pgp-Glo Assay buffer (control), ATP standards (for the construction of the calibration curve), Na_3_VO_4_ (P-gp inhibitor), verapamil (P-gp substrate, positive control), P-gp containing membranes, and MgATP in a total volume of 50 µL. Samples (2.5 µL; in the concentration range 0.7–0 mM) were added and the reaction was incubated for 1 h in 37 °C. The reaction was stopped by the addition of the detection reagent (50 µL). After 20 min of incubation, the luminescence was read.

The basal luminescence (basal ΔRLU basal) was expressed as the difference between the relative luminescence of Na_3_VO_4_ and that of the control. The luminescence (ΔRLU) of the samples was calculated as the difference between the relative luminescence of Na_3_VO_4_ and that of the samples. For the P-gp inhibitors, the specific activity of P-gp was determined using the standard ATP curve and calculating the amount of nanomoles of ATP consumed per µg of P-gp per minute. The standard ATP curve was determined by linear regression and the concentrations of ATP consumed in the samples were recalculated by the subsequent standard interpolation of RLU ATP.

### 2.5. Sensitization of MDR Cell Line

A sub-line of a human ovarian carcinoma cell line resistant to doxorubicin (HOC/ADR, A2780/ADR) was purchased from Sigma-Aldrich together with its non-resistant parental line (HOC, A2780). Both cell lines were cultivated in DMEM supplemented with 10% FBS and 1× antibiotic antimycotic solution. In addition, the cultivation medium for HOC/ADR cells was supplemented with 0.1 µM doxorubicin. The cells were cultivated and sub-cultured as described above for the HepG2 cell line. For the experiment, the cells were seeded at a concentration of 1 × 10^5^ cells/mL into the wells of 96-well plates. After 24 h, the cells were washed 3× with PBS and fresh DMEM supplemented with the flavonolignan samples (0, 10, 20 or 30 µM) was added to both cell lines. The concentration range of doxorubicin (0.3–80 µM) was then applied. After 72 h, the viability of the cells was evaluated by resazurin assay as described in Section 2.3. The fold change [22] was calculated as the ratio of IC_50_ for doxorubicin and IC_50_ for the doxorubicin co-treated with the tested sample. A fold change higher than 1 indicates a synergistic effect, while a fold change lower than 1 means an antagonistic effect.

### 2.6. Inhibition of Expression of Transporters Responsible for MDR Phenotype

For the transporter expression profiling, both cell lines—HOC and HOC/ADR were seeded into 5 cm Petri dishes at a concentration corresponding to 1 × 10^5^ cells/mL. After 24 h, the cells were washed with PBS and fresh DMEM was added. The tested concentration of the samples was 10 µM. Doxorubicin was applied at a concentration equal to IC_25_. The cells were cultivated with appropriate samples for 48 h, after which the cells were harvested using the standard procedure and centrifuged (3200× *g*; 10 min, 4 °C). The pellets were washed twice with PBS: (i) 2 mL of pre-cooled PBS (5400× *g*; 10 min, 4 °C); and (ii) 1 mL of pre-cooled PBS (10,000× *g*; 3 min, 4 °C). The pellet was resuspended in 1 mL of Trizol (Invitrogen, Carlsbad, CA, USA) and stored at −80 °C.

The RNA concentration was determined with a Quan-iT RiboGreen RNA Assay Kit (Invitrogen) using the Infinite M200 plate reader (Tecan, Männedorf, Switzerland). The cDNA synthesis was performed from 0.5 µg of total RNA with a RevertAid First Strand Synthesis cDNA Kit (MBI Fermentas, Vilnius, Lithuania). The quality of cDNA was verified by amplifying the ubiquitin C gene fragment [23].

Quantitative real-time PCR (qPCR) was performed using the ViiA7 Real-Time PCR System (Life Technologies, Camarillo, CA, USA) with a 384-well block. The reaction mixture consisted of 0.25 µL of specific 20× TaqMan Gene Expression Assay (Life Technologies); assays used in the study are listed in Table A1), 1 µL of 5× HotFIREPol Probe qPCR Mix Plus (Solis Biodyne, Tartu, Estonia), 1.75 µL of RNase free water and 2 µL of 8× diluted cDNA. The final reaction volume was 5 µL. Cycling parameters were: initial hold at 50 °C for 2 min, initial denaturation at 95 °C for 10 min, followed by 40 cycles of 95 °C for 15 s and 60 °C for 1 min. Fluorescence values were acquired after each extension phase. Samples were analyzed in duplicates, and samples with a standard deviation of duplicates >0.5 Ct were re-analyzed. A non-template control containing nuclease-free water instead of cDNA was used. The real-time PCR study followed the MIQE guidelines [24]. Relative transcript levels of the estimated genes in the cell lines were compared using the software REST 2009 (Qiagen, Hilden, Germany).

### 2.7. Data Processing and Statistical Analysis

**Figure 2:**
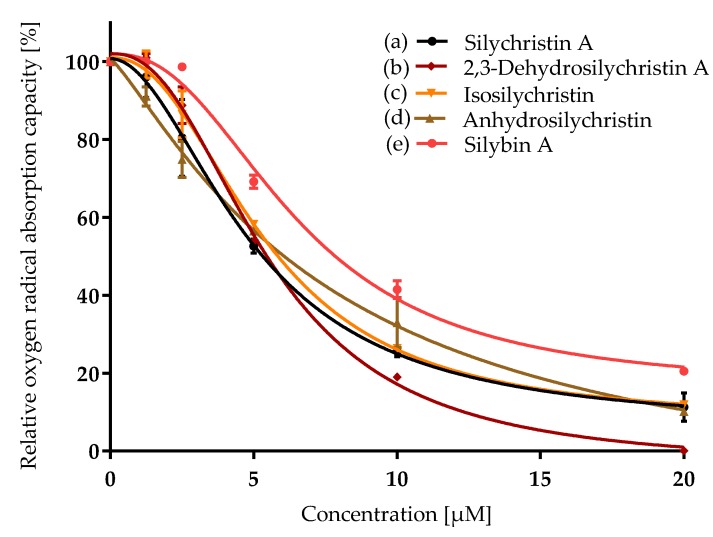
Relative oxygen radical absorption capacity (ORAC) of silychristin derivatives: (**a**) silychristin A; (**b**) 2,3-dehydrosilychristin A; (**c**) isosilychristin; (**d**) anhydrosilychristin; (**e**) silybin A. Data are presented as the average of two measurements with the respective standard error of the mean. The Ymax /Ymin values were as follows: (**a**) 100/11 ± 2; (**b**) 101 ± 1/0; (**c**) 100/12 ± 0.1; (**d**) 100/10 ± 0.1; (**e**) 100/20 ± 0.8.

**Figure 3:**
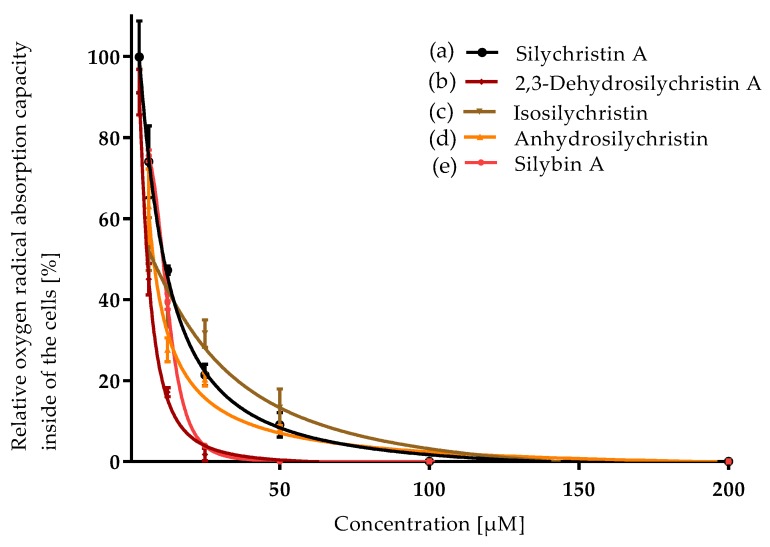
Relative oxygen radical absorption capacity of silychristin derivatives in cellular antioxidant activity assay (CAA, HepG2 cells): (**a**) silychristin A; (**b**) 2,3-dehydrosilychristin A; (**c**) isosilychristin; (**d**) anhydrosilychristin; (**e**) silybin A. Data are presented as the average of three measurements with respective standard error of the mean. The Ymax /Ymin values were as follows: (**a**) 100 ± 5/0; (**b**) 91 ± 3/0; (**c**) 54 ± 4/0; (**d**) 63 ± 5/0; (**e**) 76 ± 1/0.

**Figure 4:**
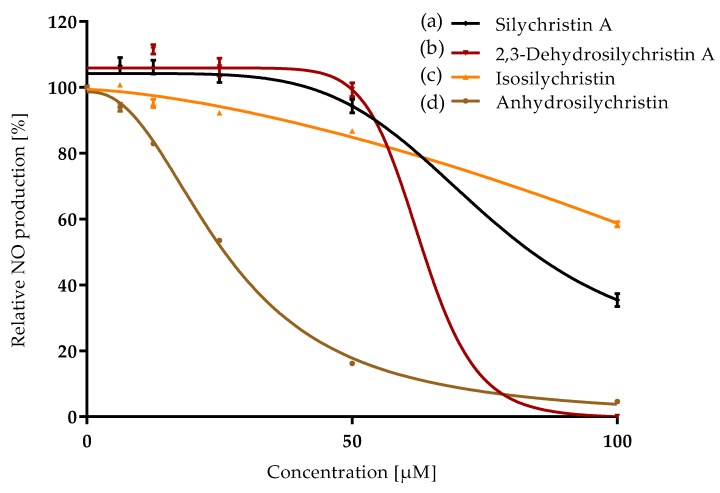
Relative nitrite oxide production as the first marker of inflammation affected by silychristin derivatives: (**a**) silychristin A; (**b**) 2,3-dehydrosilychristin A; (**c**) isosilychristin; (**d**) anhydrosilychristin. Data are presented as the average of three measurements with respective standard error of the mean. The Ymax /Ymin values were as follows: (**a**) 107 ± 2/35 ± 2; (**b**) 112 ± 1/0; (**c**) 100/58 ± 1; (**d**) 100/5 ± 0.4.

The experiments were done with the appropriate number (*n*) of repetitions, which are stated in respective figure captions. The relative activity (Figure 2, Figure 3 and Figure 4) was evaluated as a percentage according to the formula:

$$RA\;\left(\%\right)=100\frac{\mathrm{slope}\;\mathrm{of}\;\mathrm{sample}\;\mathrm{fluorescence}\;–\;\mathrm{average}\;\mathrm{slope}\;\mathrm{of}\;\mathrm{NC}}{\mathrm{average}\;\mathrm{slope}\;\mathrm{of}\;\mathrm{PC}\;-\;\mathrm{average}\;\mathrm{slope}\;\mathrm{of}\;\mathrm{NC}}.$$

IC_50_ values (Table 1 and Table 2) were determined using the software GraphPad Prism 7 (GraphPad Software, San Diego, CA, USA) —non linear regression:

Y=Bottom + (Top−Bottom)1+10^((LogIC−X)∗HillSlope).

The data are presented as the averages of the repetitions with the standard error of the mean (SEM). Statistical significance was checked with the Excel *t*-test function (two-tailed distribution, heteroscedastic type). One-way analysis of variance (ANOVA) was used, followed by Duncan’s post hoc test (*P* < 0.05), to show the differences between the groups. For ANOVA, Statistica software (Tibco Software Inc., Tulsa, OK, USA), version 12, was used.

## 3. Results

### 3.1. Antioxidant Capacity

Silychristin A and its derivatives were compared for their antioxidant capacity by the classical chemical method (ORAC) and by measurement of their radical scavenging activity inside the cells (CAA). All the samples exhibited concentration-dependent antioxidant properties in both assays (Figure 2 and Figure 3, Table 1).

In the ORAC assay, silychristin A exhibited a similar oxygen radical capacity to its derivatives, with no statistically significant differences among the respective compounds (Table 1). The antioxidant potential of silychristin A and its derivatives was compared to that of silybin (the diastereomer A was used) previously assumed to be the strongest antioxidant component of the silymarin complex. Although the IC_50_ value of silybin A was higher (6.5 ± 0.6), the *t*-test evaluated the difference as insignificant. In the cell-based assay, the results were quite different (Figure 3). 2,3-Dehydrosilychristin A and anhydrosilychristin were found to be the best antioxidant compounds followed by silychristin A, silybin A and isosilychristin. The *p*-value of the *t*-test evaluating the difference between silychristin A and 2,3-dehydrosilychristin A was 0.006, the *p*-value of the difference between silychristin A and anhydrosilychristin was 0.02. Therefore, both 2,3-dehydro- and anhydrosilychristin exhibited better antioxidant activity within the cells than silychristin A. In addition, both derivatives were stronger antioxidants than silybin A, with the *p*-value being 0.002 for 2,3-dehydro and 0.05 for the anhydro derivative (Table 1).

### 3.2. Anti-Inflammatory Properties

Inflammation was stimulated in RAW 264.7 macrophages by the addition of bacterial lipopolysaccharides. As the first signal molecule, nitrite oxide was produced by cells and detected in the assay used. Silychristin A as well as its derivatives were able to inhibit nitrite oxide production in a concentration-dependent manner (Figure 4) when added to the cultivation medium together with the lipopolysaccharide. Anhydrosilychristin was evaluated as the strongest anti-inflammatory agent followed by 2,3-dehydrosilychristin A (Table 1). Almost double concentrations of isosilychristin and silychristin A were required to achieve the same effect. The *p*-values of the *t*-test evaluating the difference between silychristin A and its derivatives (isosilychristin, 2,3-dehydrosilychristin A and anhydrosilychristin) were 0.2; 0.007 and 0.003, respectively. Thus, both 2,3-dehydro- and anhydro derivatives exhibited better anti-inflammatory activity than the parent compound.

### 3.3. Inhibition of P-Glycoprotein

**Figure 5:**
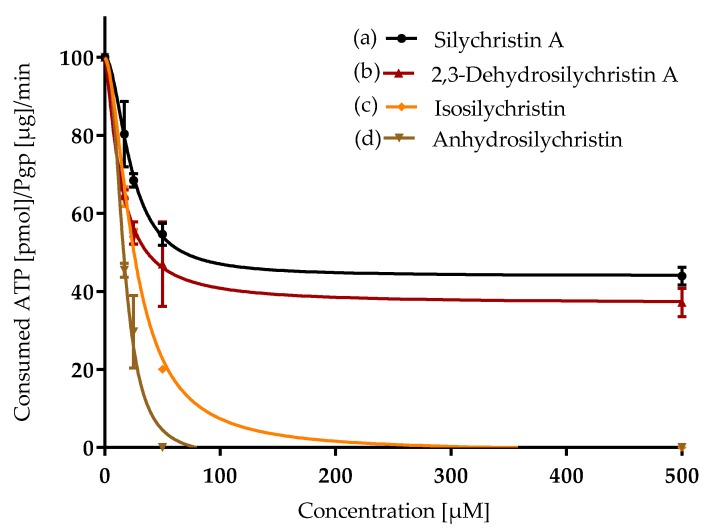
Inhibition of P-glycoprotein (transmembrane efflux pump belonging to the ATP binding cassette family) by silychristin derivatives: (**a**) silychristin A; (**b**) 2,3-dehydrosilychristin A; (**c**) isosilychristin; (**d**) anhydrosilychristin. Data are presented as the average of three measurements with corresponding standard error of the mean. The Y_max_ /Y_min_ values were as follows: (**a**) 100/44 ± 2; (**b**) 100/37 ± 2; (**c**) 100/0; (**d**) 100/0.

For the evaluation of the ability of silychristin A and its derivatives to inhibit P-gp in vitro, the isolated fractions of the membranes containing this transmembrane protein were used. This assay is based on the quantification of ATP consumed by active P-gp since inhibited P-gp does not change the ATP level. For P-gp stimulators, the ΔRLU of samples is higher than the basal ΔRLU. If the ΔRLU of the samples is equal to the basal ΔRLU, the samples have no effect on P-gp. In contrast, P-gp inhibitors exhibited a ΔRLU that was lower than the basal ΔRLU. Both silychristin A and its derivatives inhibited P-gp in a concentration-dependent manner (Figure 5). 2,3-Dehydrosilychristin A and anhydrosilychristin exhibited the lowest IC_50_ values (Table 1). Isosilychristin was a slightly weaker inhibitor than silychristin A. The *p-*value of the *t*-test comparing the difference between silychristin A and its derivatives 2,3-dehydrosilychristin A, anhydrosilychristin, and isosilychristin were 0.05, 0.1 and 0.05, respectively. Moreover, both silychristin A and 2,3-dehydrosilychristin A limited ATP consumption to only 40% of the negative control, even at the highest concentrations (100–500 µM), in contrast to anhydrosilychristin and isosilychristin, which lowered the consumption of ATP to 0 at relatively low concentrations (75 and 225 µM, respectively; Figure 5).

### 3.4. Sensitization of the Multidrug-Resistant Cell Line

In the previous section, the ability of silychristin A and its derivatives to inhibit the P-gp pump in vitro was demonstrated. Therefore, the human ovarian adenocarcinoma cell line resistant to doxorubicin was the obvious follow-up to the study under real conditions. The commercial doxorubicin-sensitive (HOC) and doxorubicin-resistant (HOC/ADR) cell lines were characterized for their sensitivity to silychristin A, its derivatives and doxorubicin. The HOC/ADR cell line was several times (approximately 300×) more resistant to doxorubicin than its parental HOC cell line (Table 2). As predicted, for both cell lines silychristin A and its derivatives were only toxic at high concentrations (>50 µM). The sensitive cell line was slightly more sensitive than the resistant cell line.

The sensitization of the resistant cell line was achieved by the addition of a single dose of silychristin A or its derivatives into the cultivation medium. After that, a concentration range of doxorubicin was applied in order to determine the IC_50_ of doxorubicin. Finally, the sensitization rate was determined as the ratio of the IC_50_ of doxorubicin to the IC_50_ of doxorubicin affected by the presence of silychristin A or its derivatives. At the lowest concentration point (10 µM), isosilychristin did not influence the HOC/ADR cell line; a mild effect was observed for the 2,3-dehydro- and anhydro derivative, and the highest sensitization was detected with silychristin A. When the concentration of silychristin A and its derivatives was doubled (20 µM), the sensitization of the HOC/ADR cell line was almost 4× more important (Table 3). Both isosilychristin and anhydrosilychristin affected the resistant cell line only slightly or not at all.

### 3.5. Inhibition of ABC Transporters Expression

Based on the finding that silychristin A and its derivatives are able to sensitize the HOC/ADR cell line, we addressed the question whether the sensitization is only caused by P-gp inhibition or by the downregulation of the corresponding gene expression. To clarify this issue, both cell lines were analyzed for the expression profiles of their ABC superfamily genes. The comparison of the RNA expression profiles of the sensitive and resistant cell lines is given in Appendix A Table A1. A low or undetectable level of expression was characteristic for 11 out of 42 ABC transporters genes (*ABCA4*, *ABCA6*, *ABCA8*, *ABCA9*, *ABCA10*, *ABCA12*, *ABCA13*, *ABCB4*, *ABCB11*, *ABCC3*, *ABCC9*, *ABCG8*). The main difference between the two cell lines was the high expression of the *ABCB1* gene (P-gp) in the resistant cell line, and no expression of this gene in the sensitive cell line. Moreover, the resistant cell line expressed a significantly higher amount of the *ABCC2* gene (BCRP protein). This overexpression was up to 400× when comparing the resistant and sensitive cell lines. In addition, there was a statistically significant overexpression of 20 other ABC genes in the resistant cell line.

After the treatment of the HOC/ADR cell line with both silychristin A and its derivatives at 10 µM, the expression profile of the ABC superfamily genes was affected considerably (Appendix A, Table A2). In each case, the *ABCA2* and *ABCF1* genes were downregulated by 25% (silychristin A and 2,3-dehydrosilychristin A)—32% (anhydrosilychristin) and 79% (anhydrosilychristin)—89% (isosilychristin), respectively. On the other hand, both the *ABCD1* and *ABCF2* genes were upregulated by each of the tested compounds by 55% (anhydrosilychristin)—78% (silychristin A) and 67% (anhydrosilychristin)—105% (silychristin A), respectively. The most significant expression profile changes were caused by anhydrosilychristin (altering the expression of 21 ABC genes) followed by 2,3-dehydrosilychristin A (altering the expression of 18 ABC genes). In contrast, both silychristin A and isosilychristin treatment only affected the expression of nine ABC genes. As described above, doxorubicin resistance is mainly caused by the over-expression of P-gp (*ABCB1* gene) and BCRP (*ABCC2* gene) in the HOC/ADR cell line. Therefore, we focused on the changes in the expression of these two genes, which were substantially affected by anhydrosilychristin. The *ABCB1* and *ABCC2* gene expression decreased by 28% and 40% respectively after the treatment with anhydrosilychristin. Isosilychristin was only able to downregulate the expression of the *ABCB1* gene by 20%. Neither silychristin A nor 2,3-dehydrosilychristin A changed the expression of the dominant ABC transporters in the HOC/ADR cells.

## 4. Discussion

The antioxidant properties of the silymarin complex and its components have been studied extensively (summarized, e.g., in [25]). The methodology for such testing was mainly based on the application of biochemical assays that have only limited relevance to the in situ conditions in tissues or the whole organism. Therefore in this study we evaluated the antioxidant properties of silychristin A, a neglected but abundant silymarin flavonolignan, and structurally related compounds by two distinct methods. An ORAC assay was performed, which is a classical biochemical method that determines the ability of the tested compounds to serve as quenchers of peroxyl radicals generated from 2,2′-azo-*bis*(2-amidinopropane) dihydrochloride (AAPH) in order to protect fluorescein against oxidation. This assay serves predominantly for the physical description of the tested compounds [26]. To reflect the biological relevance, the cellular antioxidant activity (CAA) was measured using a liver carcinoma cell line. The main difference between CAA and classical biochemical assays is that in CAA the compound must enter the cell to fulfill its role as an antioxidant. The CAA assay partially includes the aspects of both uptake and metabolism [21]. Despite the above-mentioned advantages, CAA does not provide any insight into the destiny of the tested compounds in the whole organism, including their distribution, clearance or, e.g., the ability of the tested compounds to induce the transcription of antioxidant enzymes [27].

The antioxidant activity of silymarin and its components is usually only measured by biochemical assays that demonstrate good antioxidant properties [28]. However, these antioxidant properties have been rarely demonstrated in living cells [29]. Due to difficulties with isolating the components of silymarin, the cellular antioxidant activity was only published for its main components such as silybin (and often as a diastereomeric mixture) [30], 2,3-dehydrosilybin [31], and taxifolin [32] and data on other components are still missing.

One of the recent trends in antioxidant mechanism studies shows that many nutritional antioxidants are not able to scavenge oxygen radicals in vivo. Instead, at their physiological nontoxic concentrations, they maintain nucleophilic tonus by a mechanism called “para-hormesis” leading to the activation of the transcription of the antioxidant enzymes, which leads to protection and activation of repair mechanisms [33]. This type of silymarin action was reported; specifically the ability to prevent DNA damage in human blood cells [34] or to increase the antioxidant enzyme transcription in animals [35].

Here, we report on the ability of silychristin A and its derivatives to scavenge oxygen radicals. As discussed above, each of them is a strong antioxidant, which was demonstrated by our results as well. We observed no difference either between silychristin A and its derivatives or between the derivatives and silybin A. However, quite a different situation was observed in the cellular environment. In the antioxidant activity assay; 2,3-dehydro- and anhydro derivatives were almost twice active as isosilychristin, silychristin A and silybin A. The main explanation probably lies in the differences in their structure, namely the additional double bond at position C-2, C-3 for 2,3-dehydrosilychristin and at position C-10, C-11 in the structure of anhydrosilychristin (Figure 1). This is in line with the previously reported DPPH and ABTS scavenging, reducing and anti-lipoperoxidant activity of not only 2,3-dehydrosilychristin and anhydrosilychristin, but also other 2,3-dehydroflavonolignans compared with their parent flavonolignans [1,4,36,37,38].

The anti-inflammatory activity of silymarin has been previously reported [39,40], showing its ability to affect inflammation via the suppression of the NF-κB signaling pathway and TNF-α activation. Moreover, its ability to downregulate COX-2, LOX, inducible iNOS and IL-1 was demonstrated as well [41]. However, many researchers have never taken into account the fact that silymarin is a complex mixture of several flavonolignans and flavonoids [42] and data on the anti-inflammatory activity of pure compounds are mostly [43] still missing. Here, we demonstrate that silychristin A is able to decrease an inflammatory marker (NO) in a concentration-dependent manner and its anhydro- and iso- derivatives possess almost double anti-inflammatory activity.

In general, anticancer activity is among other things connected to the ability to inhibit oncologically important transmembrane transporters. Thyroid hormone transmembrane transporter (THTT) is one such transporter, whose inhibition can reduce the aggressiveness or delay the onset of cancer [44]. Based on a THTT inhibition study with silymarin components, silychristin was evaluated as the most effective inhibitor, with an IC_50_ in the nM range [45,46]. On the other hand, the Na^+^/K^+^ ATPase inhibitors usually used in the treatment of cardiovascular diseases have recently demonstrated a beneficial effect on several cancers’ tissues [47]. The first anticancer drugs based on Na^+^/K^+^ ATPase inhibitors are in clinical trials, making this transmembrane pump an emerging target for anticancer therapy [47]. Silychristin was shown to inhibit this pump with an IC_50_ of ca. 40 µM [48]. Since the ability of silychristin to inhibit several transmembrane pumps described as cancer treatment targets has been demonstrated, the modulation of P-glycoprotein activity is another issue that should be resolved.

P-glycoprotein (P-gp) is a transmembrane efflux pump belonging to the ATP Binding Cassette (ABC) family of transporters. Its physiological function is connected to the export of cytokines, steroid hormones, ions and xenobiotics, which includes anticancer drugs [49]. The inhibition of P-gp has therapeutic importance in sensitizing multidrug-resistant (MDR) tumors to the available commercial drugs. The ability of some flavonolignans to modulate MDR was recently summarized in a comprehensive review [49]. Among them, the silymarin complex has been recognized as an inhibitor of this transmembrane pump [41,50,51] able to reverse the multidrug resistance phenotype in a doxorubicin-resistant breast cancer cell line [52]. Furthermore, silybin, as the main component of the silymarin complex, inhibited P-gp in a concentration-dependent manner [53] and reversed multidrug resistance in a small cell lung carcinoma [54]. However, to the best of our knowledge, other components of the silymarin complex have never been tested for their ability to reverse a multidrug resistance phenotype.

Two main mechanisms could be involved in P-gp activity modulation: direct inhibition of the transporter and/or inhibition of its expression. Based on our results, silychristin A and 2,3-dehydrosilychristin A modulate P-gp activity mainly via the first means. These compounds exhibited a concentration-dependent ability to decrease both the function of P-gp and decrease the resistance of P-gp overexpressing cells. In addition; neither silychristin A nor 2,3-dehydrosilychristin A affected P-gp expression. 2,3-Dehydrosilychristin A was a slightly better P-gp modulator, probably due to its highly conjugated aromatic structure [4,36]; the results were similar for the CAA assay. In contrast, anhydro- and iso- derivatives of silychristin A act as modulators of both P-gp function and P-gp expression. Both compounds decreased P-gp function in a concentration-dependent manner, sensitized the P-gp overexpressing cell line and concurrently affected the P-gp expression. The first report on the modulation of ABC transporter expression profiles by silymarin [55] suggested the upregulation of the *ABCG5*, *ABCG8* and *ABCA1* genes. We attempted to compare these data with our results. However, the first two genes were not expressed in a HOC/ADR cell line, and the last gene was unaffected or slightly downregulated (only by 2,3-dehydrosilychristin A).

## 5. Conclusions

Multidrug resistance (MDR) is one of the major upcoming challenges of the 21st century. One of the main mechanisms of MDR is the overexpression of ABC transporters such as P-glycoprotein (Pgp). Here, we demonstrated that silychristin A and its derivatives have a broader spectrum of biological activities than has been previously thought. Besides their strong antioxidant and anti-inflammatory activity, both silychristin A and its derivatives are able to inhibit P-gp in a concentration-dependent manner and thus sensitize this multidrug-resistant cancer cell line. The mode of action of silychristin A and 2,3-dehydrosilychristin A could be direct inhibition of the transporter in contrast to iso- and anhydrosilychristin, which modulate the MDR phenotype by inhibiting P-gp expression.

## Figures and Tables

**Table 1:** Concentration [µM] that halved the antioxidant, anti-inflammatory and efflux pump modulating activity (IC_50_) of silychristin and its derivatives.

Compound	ORAC IC_50_ [µM]	CAA IC_50_ [µM]	NO Production IC_50_ [µM]	P-gp IC_50_ [µM]
Silychristin A	5.4 ± 0.2 ^a^	11.4 ± 0.5 ^b,c^	65 ± 3 ^d^	21 ± 1 ^b^
2,3-Dehydrosilychristin A	5.4 ± 0.1 ^a^	6.0 ± 0.2 ^a^	36.0 ± 0.2 ^b^	15.6 ± 0.5 ^a^
Isosilychristin	6.0 ± 0.1 ^a^	13 ± 2 ^c^	59.8 ± 0.8 ^c^	25.9 ± 0.4 ^c^
Anhydrosilychristin	5.77 ± 0.05 ^a^	8.0 ± 0.7 ^a, b^	22.5 ± 0.3 ^a^	16.8 ± 0.2 ^a^

ORAC—oxygen radical absorption capacity; CAA—cellular antioxidant activity assay; NO production—anti-inflammatory activity; P-gp—inhibition of P-glycoprotein (transmembrane efflux pump). Data are presented as the concentration (µM) that halved the respective activity (IC_50_); average of three (in the case of ORAC, only two) repetitions ± standard error of the mean. Letters indicate the differences between the groups (ANOVA followed by Duncan’s post hoc test, *P* < 0.05) within one assay; the different assays were evaluated independently on each other. Statistically significant levels were denoted by different letters.

**Table 2:** Sensitization of doxorubicin-resistant human ovarian adenocarcinoma cell line by silychristin A and its derivatives expressed as IC50.

IC_50_ [µM]	HOC	HOC/ADR
Silychristin A	144 ± 2	184 ± 5
2,3-Dehydrosilychristin A	50.8 ± 0.9	68 ± 9
Isosilychristin	209 ± 3	115 ± 4
Anhydrosilychristin	45 ± 4	66 ± 3
Doxorubicin	0.022 ± 0.001	6.2 ± 0.3

Data are expressed as the concentration (µM) that halved the growth of the population (IC_50_) of the sensitive human ovarian adenocarcinoma cell (HOC) and doxorubicin-resistant HOC/ADR cell lines.

**Table 3:** Doxorubicin-sensitization rate of doxorubicin-resistant human ovarian carcinoma cells (HOC/ADR) co-cultivated with the presence of silychristin derivatives.

Compound	10 µM	20 µM
Silychristin A	(1.7 ± 0.2) ×	(4.0 ± 0.2) ×
2,3-Dehydrosilychristin A	(1.3 ± 0.1) ×	(4.8 ± 0.3) ×
Isosilychristin	(1.0 ± 0.1) ×	(1.5 ± 0.1) ×
Anhydrosilychristin	(1.5 ± 0.1) ×	(1.0 ± 0.0) ×

The sensitization rate was determined as the ratio of doxorubicin IC_50_ and doxorubicin + derivatives IC_50_.

## Abbreviations

AAPH	2,2′-azo-*bis-*(2-methylpropionamidine) dihydrochloride
ABC	ATP binding cassette
ADR	Adriamycin, trade name of doxorubicin
BCRP	breast cancer resistance protein
BHA	butylated hydroxyanisole
BHT	butylhydroxytoluen
CAA	cellular antioxidant activity
COX-2	cyclooxygenase-2
DCFH-DA	2′,7′-dichlorofluorescin diacetate
DPPH	2,2-diphenyl-1-picrylhydrazyl
DMEM	Dulbecco’s Modified Eagle’s Medium - high glucose
EMEM	Eagle´s Minimum Essential Medium
FBS	fetal bovine serum
FCR	Folin–Ciocalteu reagent
FRAP	ferric reducing ability of plasma
HepG2	human hepatocellular adenocarcinoma
HOC	human ovarian adenocarcinoma
HOC/ADR	human ovarian adenocarcinoma resistant to doxorubicin
IL-1	Interleukin-1
iNOS	inducible isoform of nitric oxide synthases
LPS	lipopolysaccharides
LOX	lipoxygenase
MDR	multidrug resistance
MEM	Essential Medium Eagle no phenol red
NF-κB	nuclear factor kappa-light-chain-enhancer of activated B cells
ORAC	oxygen radical absorption capacity
P-gp	P-glycoprotein
PBS	Phosphate-buffered saline
RAW 264.7	mouse macrophages
RLU	relative luminescence units
THTT	thyroid hormone transmembrane transporters
TNF-α	tumor necrosis factor alpha

## Appendix A

**Table A1:** Comparison of ABC (ATP-binding cassette) superfamily genes expression profiles in doxorubicin-sensitive HOC and -resistant HOC/ADR cell lines.

Gene Symbol	Assay ID	GenBank Accession No.	Gene Name	Amplicon Length (bp)	Expression Difference	*P*	Resistant versus Sensitive
Reference							
PPIA	Hs99999904_m1	NM_021130.3	Peptidylprolyl isomerase A	98			
Target							
ABCA1	Hs00194045_m1	NM_005502.3	ABC, sub-family A (ABC1), member 1	125	3.140	0.000	overexpression
ABCA2	Hs00242232_m1	NM_212533.2	ABC, sub-family A (ABC1), member 2	58	1.402	0.000	overexpression
ABCA3	Hs00184543_m1	NM_001089.2	ABC, sub-family A (ABC1), member 3	77	0.487	0.161	
ABCA4	Hs00184367_m1	NM_000350.2	ABC, sub-family A (ABC1), member 4	71	neither in HOC nor in HOC/ADR		
ABCA5	Hs00363322_m1	NM_172232.2	ABC, sub-family A (ABC1), member 5	100	4.595	0.000	overexpression
ABCA6	Hs00365329_m1	NM_080284.2	ABC, sub-family A (ABC1), member 6	83	neither in HOC nor in HOC/ADR		
ABCA7	Hs00185303_m1	NM_019112.3	ABC, sub-family A (ABC1), member 7	80	2.632	0.000	overexpression
ABCA8	Hs00992371_m1	NM_007168.2	ABC, sub-family A (ABC1), member 8	85	neither in HOC nor in HOC/ADR		
ABCA9	Hs00329320_m1	NM_080283.3	ABC, sub-family A (ABC1), member 9	145	neither in HOC nor in HOC/ADR		
ABCA10	Hs00365268_m1	NM_080282.3	ABC, sub-family A (ABC1), member 10	127	neither in HOC nor in HOC/ADR		
ABCA12	Hs00292421_m1	NR_103740.1	ABC, sub-family A (ABC1), member 12	77	neither in HOC nor in HOC/ADR		
ABCA13	Hs01110169_m1	NM_152701.3	ABC, sub-family A (ABC1), member 13	80	neither in HOC nor in HOC/ADR		
ABCB1	Hs00184491_m1	NM_000927.4	ABC, sub-family B (MDR/TAP), member 1	110	no detectable level of expression in HOC		
ABCB2	Hs00388677_m1	NM_000593.5	Transporter 1, ABC, sub-family B (MDR/TAP)	60	0.263	0.320	not affected
ABCB3	Hs00241060_m1	NM_018833.2	Transporter 2, ABC, sub-family B (MDR/TAP)	66	0.290	0.000	attenuated
ABCB4	Hs00240956_m1	NM_018850.2	ABC, sub-family B (MDR/TAP), member 4	73	neither in HOC nor in HOC/ADR		
ABCB6	Hs00180568_m1	NM_005689.2	ABC, sub-family B (MDR/TAP), member 6	60	4.339	0.000	overexpression
ABCB7	Hs00188776_m1	NM_004299.3	ABC, sub-family B (MDR/TAP), member 7	92	1.485	0.320	not affected
ABCB8	Hs00185159_m1	NM_007188.3	ABC, sub-family B (MDR/TAP), member 8	74	1.611	0.000	overexpression
ABCB9	Hs00608640_m1	NM_203444.2	ABC, sub-family B (MDR/TAP), member 9	75	1.185	0.000	overexpression
ABCB10	Hs00429240_m1	NM_012089.2	ABC, sub-family B (MDR/TAP), member 10	133	1.270	0.000	overexpression
ABCB11	Hs00184824_m1	NM_003742.2	ABC, sub-family B (MDR/TAP), member 11	63	neither in HOC nor in HOC/ADR		
ABCC1	Hs00219905_m1	NM_004996.3	ABC, sub-family C (CFTR/MRP), member 1	74	1.241	0.000	overexpression
ABCC2	Hs00166123_m1	NM_000392.3	ABC, sub-family C (CFTR/MRP), member 2	75	399.968	0.000	overexpression
ABCC3	Hs00358656_m1	NM_003786.3	ABC, sub-family C (CFTR/MRP), member 3	98	neither in HOC nor in HOC/ADR		
ABCC4	Hs00195260_m1	NM_005845.3	ABC, sub-family C (CFTR/MRP), member 4	86	1.326	0.000	overexpression
ABCC5	Hs00981089_m1	NM_005688.2	ABC, sub-family C (CFTR/MRP), member 5	68	1.619	0.000	overexpression
ABCC6	Hs00184566_m1	NM_001171.5	ABC, sub-family C (CFTR/MRP), member 6	56	0.941	0.000	attenuated
ABCC9	Hs00245832_m1	NM_020297.2	ABC, sub-family C (CFTR/MRP), member 9	70	neither in HOC nor in HOC/ADR		
ABCC10	Hs00675716_m1	NM_033450.2	ABC, sub-family C (CFTR/MRP), member 10	142	2.591	0.000	overexpression
ABCD1	Hs00163610_m1	NM_000033.3	ABC, sub-family D (ALD), member 1	101	4.417	0.000	overexpression
ABCD2	Hs00193054_m1	NM_005164.3	ABC, sub-family D (ALD), member 2	109	0.830	0.502	not affected
ABCD3	Hs00161065_m1	NM_002858.3	ABC, sub-family D (ALD), member 3	91	1.697	0.159	not affected
ABCD4	Hs00245534_m1	NM_005050.3	ABC, sub-family D (ALD), member 4	117	0.964	0.842	not affected
ABCE1	Hs01009190_m1	NM_001040876.1	ABC, sub-family E (OABP), member 1	91	1.207	0.000	overexpression
ABCF1	Hs00153703_m1	NM_001090.2	ABC, sub-family F (GCN20), member 1	69	1.706	0.000	overexpression
ABCF2	Hs00606493_m1	NM_005692.4	ABC, sub-family F (GCN20), member 2	113	1.330	0.000	overexpression
ABCF3	Hs00217977_m1	NM_018358.2	ABC, sub-family F (GCN20), member 3	61	1.792	0.000	overexpression
ABCG1	Hs00245154_m1	NM_207629.1	ABC, sub-family G (WHITE), member 1	58	2.532	0.000	overexpression
ABCG2	Hs00184979_m1	NM_004827.2	ABC, sub-family G (WHITE), member 2	92	0.467	0.000	attenuated
ABCG4	Hs00223446_m1	NM_001142505.1	ABC, sub-family G (WHITE), member 4	93	24.936	0.000	overexpression
ABCG8	Hs00223690_m1	NM_022437.2	ABC, sub-family G (WHITE), member 8	63	neither in HOC nor in HOC/ADR		

**Table A2:** Effect of silychristin A and its derivatives [10 µM] on ABC superfamily genes expression profiles in HOC/ADR cell line.

	Silychristin A			2,3-Dehydrosilychristin A			Isosilychristin			Anhydrosilychristin		
Gene Symbol	Expression Difference	*P*	Treated versus Untreated	Expression Difference	*P*	Treated versus Untreated	Expression Difference	*P*	Treated versus Untreated	Expression Difference	*P*	Treated versus Untreated
*PPIA*	1.000			1.000			1.000			1.000		
ABCA1	1.217	0.321		0.801	0.000	DOWN	0.856	0.665		0.948	0.672	
ABCA2	0.755	0.000	DOWN	0.756	0.000	DOWN	0.743	0.000	DOWN	0.675	0.000	DOWN
ABCA3	0.971	0.829		0.834	0.000	DOWN	0.755	0.336		0.686	0.000	DOWN
ABCA5	0.971	0.829		0.965	0.675		0.864	0.499		0.822	0.341	
ABCA7	1.220	0.172		1.241	0.000	UP	1.142	0.166		1.054	0.846	
ABCB1	0.932	0.321		0.863	0.654		0.796	0.000	DOWN	0.722	0.000	DOWN
ABCB2	0.980	0.657		0.825	0.347		0.753	0.166		0.546	0.000	DOWN
ABCB3	1.007	0.828		0.902	0.832		0.962	0.669		0.693	0.000	DOWN
ABCB6	0.799	0.000	DOWN	0.707	0.000	DOWN	0.758	0.166		0.557	0.000	DOWN
ABCB7	0.855	0.321		0.735	0.157		0.769	0.166		0.820	0.174	
ABCB8	0.833	0.000	DOWN	0.811	0.328		0.953	0.665		0.554	0.000	DOWN
ABCB9	0.924	0.657		0.743	0.000	DOWN	0.848	0.000	DOWN	0.590	0.000	DOWN
ABCB10	0.883	0.657		0.774	0.157		0.626	0.166		0.568	0.000	DOWN
ABCC1	0.917	0.321		0.774	0.000	DOWN	0.711	0.166		0.654	0.000	DOWN
ABCC2	1.025	0.828		0.869	0.675		0.761	0.166		0.596	0.000	DOWN
ABCC3	6.375	0.172		5.600	0.000	UP	4.569	0.170		3.812	0.174	
ABCC5	0.895	0.321		0.773	0.000	DOWN	0.697	0.166		0.615	0.000	DOWN
ABCC6	1.094	0.321		0.861	0.675		0.841	0.499		1.159	0.000	UP
ABCC10	1.006	0.828		0.752	0.000	DOWN	0.752	0.000	DOWN	0.628	0.000	DOWN
ABCD1	1.775	0.000	UP	1.664	0.000	UP	1.700	0.000	UP	1.552	0.000	UP
ABCD2	2.925	0.000	UP	4.374	0.000	UP	0.827	0.497		4.267	0.329	
ABCD3	0.957	0.657		0.793	0.504		0.293	0.166		0.964	0.331	
ABCD4	2.146	0.172		0.752	0.000	DOWN	1.822	0.000	UP	1.686	0.174	
ABCE1	1.854	0.000	UP	1.811	0.157		2.080	0.000	UP	2.234	0.329	
ABCF1	0.161	0.000	DOWN	0.182	0.000	DOWN	0.114	0.000	DOWN	0.208	0.000	DOWN
ABCF2	2.053	0.000	UP	2.028	0.000	UP	1.753	0.000	UP	1.672	0.000	UP
ABCF3	1.116	0.172		0.464	0.000	DOWN	0.993	0.669		0.799	0.000	DOWN
ABCG1	1.448	0.172		0.844	0.000	DOWN	0.900	0.665		1.760	0.000	UP
ABCG2	0.891	0.657		0.804	0.000	DOWN	0.922	0.830		0.716	0.000	DOWN
ABCG4	1.854	0.000	UP	1.533	0.000	UP	1.513	0.166		1.583	0.000	UP
